# Special Issue “Recent Advances of Novel Pharmaceutical Designs for Anti-Cancer Therapies, 3rd Edition”

**DOI:** 10.3390/ijms27198511

**Published:** 2026-09-24

**Authors:** Bernhard Biersack

**Affiliations:** Organic Chemistry Laboratory, University of Bayreuth, Universitätsstrasse 30, 95440 Bayreuth, Germany; bernhard.biersack@yahoo.com

In recent years, there has been some impressive progress made in cancer research in terms of sophisticated new small-molecule drugs, particularly targeted drugs, and of immune, cell, and gene therapies, which have saved and prolonged the lives of many cancer patients [1]. Nevertheless, according to the WHO, cancer is still the second-highest cause of death (after cardiovascular diseases), with 26,000 cancer deaths every day and a predicted increase of new annual cancer cases from ca. 20.6 million currently to almost 35 million in 2050 [2]. Intensive research efforts are required to solve persistent problems of cancer therapy such as intrinsic or acquired drug resistance and missing treatment options in hitherto incurable cancers [3,4].

This Special Issue, entitled “Recent Advances of Novel Pharmaceutical Designs for Anti-cancer Therapies, 3rd Edition”, was launched in summer 2024. It follows up on the successful articles “Recent Advances of Novel Pharmaceutical Designs for Anti-cancer Therapies” (twelve articles) and “Recent Advances of Novel Pharmaceutical Designs for Anti-cancer Therapies 2.0” (nine articles) previously published as Special Issues by the *International Journal of Molecular Sciences* [5,6]. Once again, the 3rd edition of this series of Special Issues can present excellent developments in cancer research associated with drug and target discovery, contributed by an international board of cancer scientists. At the moment of the preparation of this editorial, a 4th edition of this Special Issue series has already been launched, welcoming new contributions to this field [7].

Immunotherapies have become a valuable treatment of various solid tumors; however, several resistance mechanisms can limit their clinical application [8]. Nevertheless, it has been shown that renal cell carcinoma (RCC) patients can profit distinctly from an adjuvant therapy with the immune checkpoint inhibitor (ICI) pembrolizumab [9]. In their review article, Gluba-Sagr and coworkers described the current clinical status of ICIs for the therapy of clear cell RCC (ccRCC), the reasons for ICI resistance in the tumor microenvironment (TME) of ccRCC (e.g., an immunosuppressive environment), as well as suitable strategies and treatment options to overcome ICI-associated resistance mechanisms such as combination with VEGFR inhibitors and the targeting of notorious TME factors (tumor-associated macrophages, regulatory T cells, cytokine signaling) [10]. Thus, a thorough understanding of the TME can lead to optimized RCC treatments and the overcoming of ICI resistance mechanisms.

Hypoxia (oxygen starvation by insufficient blood supply) in the TME, as well as considerable oxidative stress, were identified as important factors responsible for the progression of breast cancers [11]. ERO1α (endoplasmic reticulum oxidoreductin 1 alpha) is a protein that regulates redox homeostasis in the endoplasmic reticulum (ER) and its expression was found to be upregulated in aggressive cancers under hypoxic conditions, thus promoting cancer progression and spread [12,13]. Aljofan and coworkers provided a review article covering the role of ERO1α in breast cancers. Links between ERO1α and pathways such as VEGF-A (angiogenesis) and PD-L1 (immune evasion) were discussed, and progress in the development of ERO1α inhibitors were outlined. These inhibitors might lead to the development of clinically approved targeted drugs against breast cancers with overexpressed ERO1α in the future [14].

In addition to these sound review articles, several notable research papers were published in this Special Issue, which reported new anti-cancer drugs and combination treatments. The synthesis of new anti-cancer active compounds is a prolific field of research generating numerous promising drug candidates every year. Multicomponent reactions are an eco-friendly time- and cost-effective way to synthesize anti-cancer active compounds [15]. The three-component Mannich reaction was frequently applied to prepare new active derivatives with biological activities [16]. Strzelecka and coworkers used 1,2,4-triazoles connected with a 4,6-dimethylpyridine ring via amide bonds for the synthesis of new Mannich bases by reaction with *N*-phenylpiperazines and formaldehyde. The authors identified one Mannich base derivative with cytotoxic activity against gastric and colon cancer cells, which was accompanied by a low toxicity to non-malignant intestinal epithelium cells, as well as distinct pro-apoptotic effects, the reduction in IL-6 expression, and the inhibition of the notorious drug efflux pump P-gp in colon cancer cells [17]. IL-6 plays a crucial role in inflammatory and immunological processes in colorectal cancers, while the P-gp transporter has become a valuable target in multidrug-resistant cancers [18,19]. Hence, the identified 1,2,4-triazole Mannich base has potential as a relatively non-toxic modulator of tumor immunology in the TME and as an enhancer of the anti-cancer activities of P-gp substrates, which include several approved and widely applied anti-cancer drugs.

Matrix metalloproteinases (MMPs) are overexpressed in many aggressive tumors because they are required to cleave components of the extracellular matrix (ECM), thus enabling tumor growth, invasion, and migration [20]. The importance of MMP-9 inhibitors for the therapy of various cancers has already been reviewed in the 2nd edition of this Special Issue series [21]. Currently, Bardaweel and coworkers have reported five new MMP-12 inhibitors (two cinnamoyl/4-chlorocinnamoyl amides of 4-aminobenzophenone, two feruloyl amides of 3- and 4-aminobenzophenone, and one 2-chlorobenzamide of tryptamine), which exhibited antiproliferative, pro-apoptotic, and antimigratory activities against lung cancer cells, along with cell cycle arrest and colony formation inhibition [22]. Since scientific reports about the role of MMP-12 in cancers are relatively rare, this research article adds to the current knowledge of MMPs in lung cancers.

Serine/threonine-specific protein kinases play a pivotal role in cancer-associated processes and thus have become an attractive drug target for the design of promising new anti-cancer drugs [23,24]. The PIM-1 serine/threonine kinase has been found to be an important factor for the progression and resistance of various cancers [25]. Wińska and coworkers identified a 1-propan-2-one-substituted 4,5,6,7-tetrabromobenzimidazole (TBBi) derivative with cytotoxic and pro-apoptotic activity against acute lymphoblastic leukemia (ALL) and breast cancer cells. This TBBi compound inhibited recombinant PIM-1 kinase in vitro, as well as PIM-1 kinase activity inside cancer cells. It was concluded that PIM-1 inhibition by TBBis can be a valuable treatment option for the therapy of leukemias such as ALL [26].

Cyclin-dependent kinases (CDKs) are serine/threonine-specific protein kinases regulating cell cycle progression and thus play a key role in cancer development and maintenance, which has led to the design of several promising CDK inhibitors for cancer therapy [27]. In particular, some potent CDK4/6 inhibitors have emerged and have already been clinically approved for the treatment of hormone receptor-positive breast cancer [28]. Moreover, the repurposing of anti-depressants for cancer therapy has become a prolific field of anti-cancer drug research [29]. Goh and coworkers reported remarkable synergy effects of the CDK4/6 inhibitor abemaciclib in combination with the tricyclic anti-depressant desipramine in colon cancer cells (in terms of induction of cell death and cell cycle arrest) and in a colon cancer xenograft model (in terms of colon tumor growth inhibition in mice) at safe and well-tolerated drug doses [30]. These promising results underscore the great potential of combination therapies with CDK4/6 inhibitors and anti-depressants, and research efforts are currently underway to elucidate the underlying mechanisms of the combination of abemaciclib with desipramine.

The checkpoint kinase CHK1 is another important serine/threonine-specific protein kinase. The ATR/CHK1 axis plays a vital role in DNA damage response and presents a promising target for anti-cancer drug development [31]. PARP enzymes were also described as important regulators of DNA damage response and are critically involved in replication stress, which led to the design of potent PARP inhibitors (PARPis) as anti-cancer drugs [32]. Rogalska and coworkers reported the effects of the combination of the CHK1 inhibitor MK-8776 with the PARPi olaparib in hepatocellular carcinoma (HCC) cells and in an olaparib-resistant ovarian carcinoma PDX model. MK-8776 plus olaparib exhibited synergistic activities against HCC cells in terms of tumor cell viability reduction and induction of oxidative stress and DNA damage. Moreover, this combination therapy inhibited the growth of olaparib-resistant ovarian carcinoma in vivo and prevented the formation of liver metastasis while showing no hepatotoxicity [33]. Hence, a combined CHK1 and PARP inhibition can become a suitable and safe therapeutic strategy for HCCs and PARP inhibitor-resistant ovarian carcinomas.

Targeted radionuclide therapy (TRT) is based on radiopharmaceuticals (e.g., ^177^Lu complexes) that are chemically modified with functional moieties (e.g., peptides), enabling the selective targeting of tumor cells [34]. It has become a valuable treatment option for various cancer diseases including prostate cancer and neuroendocrine cancers because of its improved safety and efficacy in comparison with common radiation therapy [35]. The G-protein-coupled receptor CCK2R (cholecystokinin-2 receptor) is overexpressed in several cancers, and peptide ligands of this receptor can be applied for drug targeting, especially for radiopharmaceuticals [36]. Chiang and coworkers reported the synthesis of highly stable new ^111^In- and ^177^Lu-labeled peptide–radionuclide conjugates (INER-PP-F11N) for the treatment of CCK2R-overexpressing cancers. The conjugates exhibited a higher uptake of CCK2R-positive tumors and a reduced kidney accumulation when compared with the reference peptide conjugate PP-F11N, as well as a considerable growth inhibition of CCK2R-positive tumors in treated mice [37]. The development of INER-PP-F11N marks another milestone in improving targeted and personalized tumor therapies based on radionuclides.

Overall, the 3rd edition of this Special Issue series has become another valuable collection of outstanding review and research articles about the current state and new developments of anti-cancer drugs and therapies in treating various perilous cancer diseases including leukemia (ALL), breast cancer, ovarian cancer, hepatocellular carcinoma, colon cancer, lung cancer, renal cell carcinoma, and CCK2R-positive cancers (Figure 1). The presented anti-cancer drug targets included eminent factors and mechanisms of the TME (e.g., tumor immunology/immune checkpoints and interleukins, hypoxia, matrix metalloproteinases) as well as vital proteins (e.g., protein kinases, receptors, drug efflux pumps) and modes of action (e.g., cytotoxicity and apoptosis, cell cycle arrest, migration) of the investigated tumor cells. New and known small-molecule drugs, as well as sophisticated protein/antibody- or peptide conjugate-based drugs, were described and discussed for their anti-cancer potential. For more details, readers are cordially invited to explore the full length open access articles available from the journal and from the Special Issue website below.

## Figures and Tables

**Figure 1:**
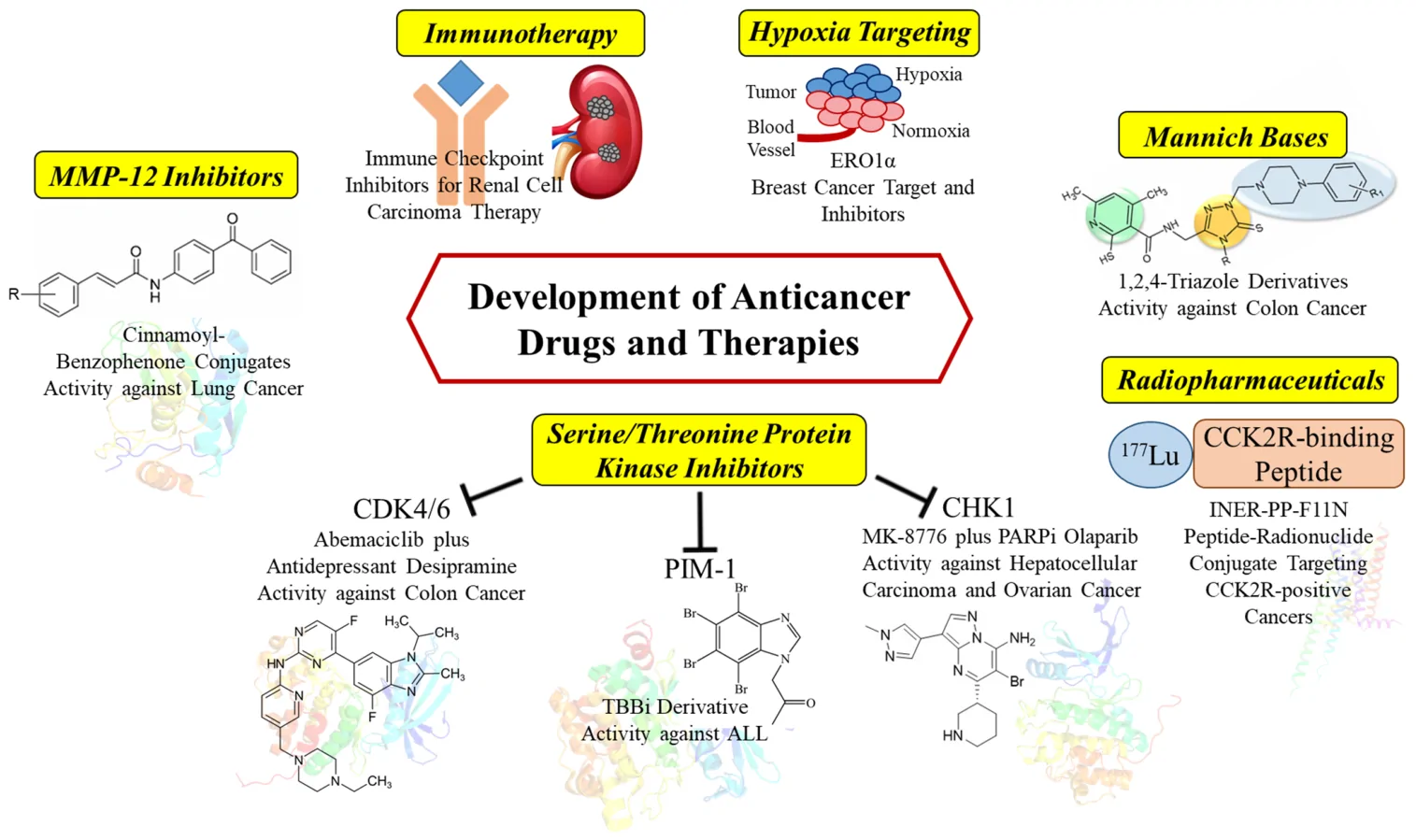
Topics covered in this Special Issue, “Recent Advances of Novel Pharmaceutical Designs for Anti-cancer Therapies, 3rd Edition”, include two review articles about immunotherapy in renal cell carcinoma [10] and the role of ERO1a as a target in breast cancers [14], as well as six research articles about the development of new anti-cancer active Mannich bases [17] and MMP-12 inhibitors [22], the identification of a new TBBi-based PIM-1 serine/threonine-specific protein kinase inhibitor with activity against leukemia (ALL) [26], the effects of known serine/threonine-specific protein kinase inhibitors (i.e., CDK4/6 and CHK1 inhibitors abemaciclib and MK-8776, respectively) in combination with other drugs (anti-depressant or PARPi) [30,33], and the design of improved CCK2R-targeting peptide–radionuclide conjugates [37].
